# Medicalisation and Overdiagnosis: What Society Does to Medicine

**DOI:** 10.15171/ijhpm.2016.121

**Published:** 2016-08-31

**Authors:** Wieteke van Dijk, Marjan J. Faber, Marit A.C. Tanke, Patrick P.T. Jeurissen, Gert P. Westert

**Affiliations:** Celsus Academy for Sustainable Healthcare, and Scientific Institute for Quality of Healthcare, Radboud Institute for Health Sciences, Radboud University Medical Center, Nijmegen, The Netherlands.

**Keywords:** Medicalisation, Overdiagnosis, Society

## Abstract

The concept of overdiagnosis is a dominant topic in medical literature and discussions. In research that targets overdiagnosis, medicalisation is often presented as the societal and individual burden of unnecessary medical expansion. In this way, the focus lies on the influence of medicine on society, neglecting the possible influence of society on medicine. In this perspective, we aim to provide a novel insight into the influence of society and the societal context on medicine, in particularly with regard to medicalisation and overdiagnosis.

## Introduction

The concepts of overdiagnosis and medicalisation are related, but not the same.^1^ Overdiagnosis can be defined as: “[t]he detection of abnormalities that are not destined to ever bother us” or “that will never cause symptoms or death.”^2^ By medicalisation we mean: “defining a problem in medical terms, usually as an illness or disorder, or using a medical intervention to treat it.”^3^ Medicalisation is not by definition a negative development, medicalising certain situations has had tremendous benefits.^4^ This in contrast to overdiagnosis, in which the ‘over’ inherently indicates excess.^5^ Both overdiagnosis and medicalisation result in more people receiving a medical diagnosis. However, the origin of this expansion differs. Medicalisation often concerns new diagnoses, based on a widened understanding of human situations that usually benefit from medical involvement. It, thus, widens the boundaries of medicine. Overdiagnosis, instead, starts inside of medicine, addressing the problem of people receiving a unbeneficial diagnosis.^1,6^ Both processes do not just happen. Medicalisation is created by a specific set of cultural and social conditions, and can be pushed by forces in and outside of medicine.^3,7^ Overdiagnosis can also be influenced by cultural and societal conditions, yet the current discussion focuses primarily on forces inside medicine. In recent years, both concepts are becoming more alike, and differences are not always clear.^1^

However, *how* the process of medicalisation takes place is not resolved with these definitions, nor is the possible influence of society on medicine, medicalisation, and overdiagnosis addressed. In this perspective, we illustrate how societal developments can result in both medicalisation and overdiagnosis. We need to bear in mind that society often has a interest in more medicine for its inhabitants, to help its inhabitants but also to depoliticise social problems.^8^ This will help us get a better grasp on ‘how medicalisation influences medicine and overdiagnosis.’

## Medicalisation as a Sociological Concept

Research after overdiagnosis often frames medicalisation as the result of forcing unnecessary medicine into people’s lives. Although this fits remarkably well with Ivan Illich’ well-known view on medicalisation and iatrogenic harms –introduced in his ground-breaking Medical Nemesis from the 1970s^9^ – it also pushes the discussion towards ‘what medicine does to people.’ This can easily result in a view of patients as the passive recipients of medicine’s well-meant mission creep. By doing so we lose track of how medicalisation in its turn is also changing - in fact shaping - modern medicine.

While the historic perspective on medicalisation blamed medical imperialism for clinical, social, and cultural iatrogenisis,^9^ contemporary analysts emphasize that medicalisation is context dependent, involving actors such as the pharmaceutical industry, the media, consumers and/or, biotechnology.^3^ Doctors are not necessarily amongst the drivers of this process and sometimes fundamentally act as gatekeepers.

Nonetheless, research often focuses on one dominant cause, like that after disease mongering blaming the pharmaceutical industry for selling sickness and pushing medicalisation.^10^ Sociology has a broader perspective and approaches medicalisation as a social process, influenced by many actors.^3^ Society’s norms and values develop at a continual pace, influencing all of us in our perception of health, what constitutes a medical problem, and who should be consulted when experiencing a problem that can be perceived as medical.^10-12^ As a result the definition of health and illness develops. Therefore, medicalisation should rather be regarded as a continuum than as a dichotomy, as problems can be regarded more or less as medical and can be treated more and less intensive. This is an addition to traditional definitions of medicalisation, which disregard the extent to which a situation or condition is medicalised.

## Societal Implications of Overdiagnosis

When discussing overdiagnosis and its consequences the underlying assumption seems to be that diagnosing is an objective and strictly medical procedure, which physicians would accomplish beautifully if they would only have the perfect knowledge. Besides the conceptual omissions in this interpretation of overdiagnosis,^13^ it is also untrue: disease and illness are not merely given biological facts but social constructions as well.^14,15^ The discussion whether disease can be defined entire value-free or is unavoidably value-laden remains unsettled, although all agree that values do have a role in the perception of disease.^16^ Societal actors such as governmental agencies can press their values on the health system by policy-making or prioritising certain diseases or treatments.

An example of how ‘disease’ is more complex than a biological fact is the current scare for and treatment of hypertension. Firstly, this condition is in itself nothing more than a diagnosis based on a cut-off point. In the end, this diagnosis solely serves to identify a risk factor for cardiovascular conditions, such as heart attack and stroke.^10,17^ Secondly, in the focus on lowering this risk with pharmaceutical treatment we may overlook that hypertension is one of several risk factors, and, even more important, can be lowered or prevented with lifestyle change.^18^ By looking at hypertension from a purely medical view, other risk factors such as an unhealthy diet, obesity, and physical inactivity are easily overlooked. Furthermore, these risk factors are strongly related to socio-economic determinants such as education and occupation, with the result that those that lose out economically are also losing out healthwise. Focussing on pharmaceutical quick-fixes instead of addressing the underlying socio-economic problems possibly leads to more inequality, both globally^19^ and nationally. As Conrad and Barker put it: “*it seems that we have a social predilection toward treating human problems as individual or clinical – whether it is obesity, substance abuse, learning difficulties, aging, or alcoholism- rather than addressing the underlying causes for complex social problems and human suffering.*”^15^ This does not mean that medicalising a situation rules out simultaneous action on its social and political determinants. Physicians can be amongst the most passionate proponents of societal change for some of the medical problems they face in their practices, such as stricter regulations for tobacco industry, sugar-taxes on beverages and calls for obesity prevention.^20-22^ Nonetheless, by our tendency to seek medical solutions for social problems, we medicalise social issues such as inequality, deviance and abnormality and locate the sources and solution of these problems increasingly on the individual level.^15^

## Medical Solutions for Societal Questions: Three Examples

In the previous paragraphs, we have shown that medicalisation is more than the result of objective choices made within medicine. Here we illustrate this with three examples in which societal influences affect the use of medical resources: the care for mentally disabled, the increased attention for treatment of Alzheimer disease (AD) and mild cognitive impairment (MCI) for the elderly, and the medicalisation of childbirth. We chose these three examples to illustrate how societal developments and medicine can interact. Comparable developments are detectable in all areas of healthcare. We choose examples that differ with regard to the influence of medicalisation and overdiagnosis. We did so to illustrate that although they are often related; they are not mutually dependent and can occur separately.

Mental disability can prevent people from full participation in society. Those with severe mental disability often have the mental abilities of a young child and cannot live unassisted. Mentally retarded people are able to function more independently but often require assistance in various living areas. The number of mentally disabled has not increased over the last decade in the Netherlands and the division of those with severe mental disability (IQ score below 50), moderate mental disability (IQ scores between 50 and 70) and those deemed mentally retarded (IQ scores between 70 and 85) was stable over this period.^23^ Overdiagnosis seems not to be present in this case. Nonetheless, the costs for care and assistance for people with mental disabilities has increased with 7.3% annually, in the period 2007-2011.^24^ The increase in costs can only partly be ascribed to increases in wages and is for the larger part the result of increasing demand among people with moderate mental disability or mental retardation.^24^ The number of beds for inpatient care did increased with 3.4% annually during this same period.^25^ Recent policy adjustments are aimed at interrupting this trend, but effects are not observable yet. What is happening here? The threshold for receiving institutional care has lowered towards higher IQ scores.^23^ What does this imply? Can the mentally impaired not hold pace with the increasing complexities of modern society? Is this supply-induced demand, resulting from provider interest? Do we lose our ‘patience’ with slow adaptors? Or is more institutional care the medicalised answer of a society that ultimately values economic efficiency over inclusiveness? The lowering of indication thresholds is probably not solely driven by medical professionals but by societal demand as well.

The second example shows that the impact of medicalisation may differ as a result of local cultural context. Due to the aging populations of most western countries the number of people that will receive the diagnoses AD and MCI is increasing. At the same time, AD and especially MCI are not uncontested as they might medicalise normal aging. A striking illustration is the discussion in the United Kingdom about early detection of AD. Governmental policy stimulates doctors and practices to increase their number of dementia diagnoses, to benefit patients with earlier diagnosis and better treatmen.^26^ Doctors disagreed, stating that earlier diagnosis has no proven benefit, MCI does not necessarily result in dementia and overdiagnosis looms.^27,28^ This is an example of doctors acting as gatekeepers to prevent further medicalisation and overdiagnosis.

Furthermore, what distinguishes MCI or even AD from ‘normal’ cognitive aging is still unclear after a century of research.^29^ This further emphasizes how disease thresholds and diseases are socially constructed.^30^ More poignant is how cultural norms and contextual factors influence how medicalisation takes place. The Dartmouth atlas shows the percentage of people over 65 filling at least one prescription of dementia medication in 2010 in the United States. Percentages differ between regions, ranging from 3.7 to 17.1%^ [1] ^. This reveals large practice variation within the United States. Striking as this is, the figure conceals how high a percentage as low as 3.7% might be from another cultural perspective. In the Netherlands, 1.2% of people over 65 used dementia medication at least once in 2013^ [2] ^. The prevalence of dementia is slightly higher in the Netherlands than in the United States.^31^ Overdiagnosis does not seem to be present here, but over- or undertreatment may be at stake.^5^ This cannot be determined here. What we do know is that people with advanced AD more often receive long term care in the Netherlands than they do in the United States.^32^ It is not obvious whether use of pharmaceuticals or intuitional care constitutes of more medicalisation as both use medical language, medical assistance and a share of the healthcare budget. A highly relevant but understudied research question is how overdiagnosis and medicalisation drive different treatment options across different countries and communities.

Childbirth is one of the examples where medicalisation has had significant benefits, diminishing the chances of maternal and child mortality. Access to medical care in case of complications during pregnancy or birth is essential. However, there is an ongoing debate whether nowadays the standard care for pregnancy in most western countries involves too much medicine and is beyond the point of provable benefit.^33^ Childbirth is an example of how medicalisation can be regarded as a continuum: Less medicalised assistance in pregnancy and birth, as provided by a midwife, differs in intensity of medical intervention from gynaecological and surgical interventions. Midwife assisted birth can, thus, be considered a less medicalised situation.

A well-established example of increasing medicalisation for childbirth is caesarean section rates (CSR). It is known that CSR vary greatly between countries and that these rates increased in the last decennia in many countries.^34^ The World Health Organization (WHO) regards a CSR between 10% and 15% ideal and states that no reduction in maternal and newborn mortality outcomes at the population level are found at a CSR higher than 15%.^35^ Higher percentages, at least on group level, could thus, be interpreted as an indication of overdiagnosis. Most western countries exceed this percentage, which ranged in Europe from 14.8% in Iceland to 52.2% in Cyprus in 2010.^36^ In the United States, 31.8% of live births was delivered by CSR in 2007.^34^ The choice for CSR depends on many variables on the individual and health system level.^37,38^ The percentage of women preferring CS varies between countries, but never exceed 14%.^39^ In the Netherlands, the CSR is 17.0%, the third lowest level in Europe.^36^ Nonetheless, the percentage of homebirths is decreasing, while the use of epidurals increases and the CSR rises, indicating that childbirth is in the process of being further medicalised in the Netherlands as well.^40^ This example illustrates that many factors can contribute to medicalisation, on several levels.

## The Dual Relationship Between Overdiagnosis and Medicalisation

The three examples illustrate that the societal context influences medical decision-making as well. We illustrated how medicalisation can occur on its own regard and how it can lead to overdiagnosis. Coleman’s boat shaped scheme provides a nice metaphor to illustrate this (Figure). Crucial to this metaphor is the relation between macro and micro developments. Consider medicalisation as a macro condition: a set of societal norms and values, influencing us all. This influences behaviour and expectations on the micro level, in the consultation between doctor and patient, allegedly resulting in more diagnoses and treatments. As a macro result, an increasing use of healthcare and possibly overdiagnosis is detected. For example: within a more medicalised society, acceptance of forgetfulness amongst the elderly decreases. As a result elderly people grow more conscious of their forgetfulness and consult their physicians more often and probably earlier than they would have done otherwise, resulting in an increasing number of diagnoses and prescriptions.

**Figure:**
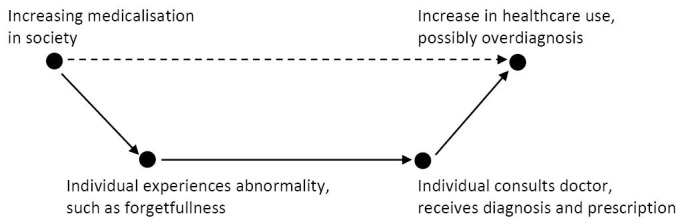
Coleman’s Boat.^41^

The metaphor stops here, but we suggest adding another relation. An extra dotted arrow should be drawn from macro result to macro condition, indicating that a macro result in turn also influences the macro condition. In this case, overdiagnosis further enhances medicalisation. The suspected mechanism behind this lies in the increasing societal consciousness of conditions and its treatments, decreasing the individual and societal tolerance to endure everyday complaints.

## To Conclude

In this perspective, we argue that instead of solely a result of medicine, medicalisation and overdiagnosis consists of social cultural processes that take place both in *and* outside medicine. Medicalisation entails a complex set of drivers, including interests, existing institutional rules, and the way society defines ‘disease’ and ‘normality.’ Both overdiagnosis and medicalisation push healthcare consumption and lead to additional healthcare costs. Medicalising a situation can improve the health status of new patients. The question remains whether the possible benefits are worth the individual suffering, iatrogenic damage or social exclusion that can also be the result of it. To answer this question, medicalisation and overdiagnosis need to be analysed in a broader context, also taking into account societal aspects.

Medicalisation should be perceived as a societal phenomenon; as a multiplayer game, involving societal forces, institutional rules and stakeholder interests. Medicalisation and overdiagnosis hold an ambivalent relationship. Medicalisation partly follows from overdiagnosis in the doctor’s office. At the same time, due to increasing medicalisation at the macro level overdiagnosis on the micro level is induced. Societal developments and values, thus, influence the practice of medicine. This is a relationship we all should be conscious of, because in the end, there are limits to what medicine can improve both on an individual and a societal level.

## Ethical issues

Not applicable.

## Competing interests

Authors declare that they have no competing interests.

## Authors’ contributions

First author conceptualised the article, all others provided substantive input and suggestions on subsequent versions.

## Endnotes


[1] http://www.dartmouthatlas.org/data/map.aspx?ind=245 (accessed on May 1, 2015).

[2] Own calculations, based on https://www.gipdatabank.nl/default.asp (accessed on May 1, 2015).

